# Characterization of trazodone metabolic pathways and species-specific profiles

**DOI:** 10.3389/fphar.2025.1636919

**Published:** 2025-09-30

**Authors:** Vanessa Petrucci, Patrizia Dragone, Marcello C. Laurenti, Laura Oggianu, Volha Zabela, Agnese Cattaneo

**Affiliations:** ^1^ Angelini Pharma S.p.A., Rome, Italy; ^2^ Angelini Pharma France SASU, Rueil-Malmaison, France

**Keywords:** trazodone metabolism, cytochrome P450, drug interactions, pharmacokinetics, drug metabolism

## Abstract

**Background:**

Trazodone, an antidepressant indicated for the treatment of major depressive disorder (MDD), undergoes complex metabolism involving multiple cytochrome P450 (CYP) isoforms. Despite its widespread clinical use, there is limited contemporary research on trazodone biotransformation, particularly regarding interspecies differences mediated by CYP enzymes. This study investigates the hepatic metabolic stability, pathways, and inhibitory effects of trazodone and its metabolites.

**Methods:**

The hepatic metabolic stability of trazodone was evaluated in cryopreserved hepatocytes from human, mouse, and rat sources. Metabolic profiling was conducted using human and rat liver microsomes, hepatocytes, and rat plasma to identify primary and secondary metabolites. CYP isoforms involved in trazodone metabolism were identified through selective CYP inhibitors and recombinant enzymes. Additionally, the inhibitory potential of trazodone and m-chlorophenylpiperazine (mCPP) —the only pharmacologically active metabolite—on CYP enzymes was assessed in both human and mouse hepatocytes.

**Results:**

Trazodone demonstrated significant interspecies differences in intrinsic clearance rates, with human hepatocytes exhibiting the slowest conversion, suggesting prolonged exposure and potential for stable plasma levels. CYP3A4 was identified as the primary enzyme mediating trazodone metabolism, particularly in the formation of mCPP while CYP2D6, CYP2C19, and FMOs contributed to the formation of other major, inactive metabolites such as M9 and M2.

**Conclusion:**

CYP3A enzymes were identified as the primary mediators of trazodone metabolism, particularly for the formation of its only active metabolite, mCPP, with additional contributions from CYP2D6, CYP2C19, and FMOs to other pathways. Interspecies differences were most pronounced for CYP3A-mediated metabolism and hepatic clearance, reinforcing the importance of human-specific models to characterize trazodone’s pharmacokinetics. These findings advance the understanding of its biotransformation and support its optimized clinical use in major depressive disorder—particularly in complex therapeutic regimens and genetically diverse populations.

## 1 Introduction

Trazodone, first synthesized and introduced in the 1970s, is a widely prescribed antidepressant classified as a serotonin receptor antagonist and reuptake inhibitor (SARI). It is approved by several European regulatory agencies for the treatment of major depressive episodes, including those with co-occurring anxiety symptoms (Stahl, 2009; Fagiolini et al., 2012; Khouzam, 2017). Its pharmacological profile combines serotonin reuptake inhibition with potent antagonism at 5-HT2A receptors, distinguishing it from selective serotonin reuptake inhibitors (SSRIs), serotonin–norepinephrine reuptake inhibitors (SNRIs), and tricyclic antidepressants (TCAs) (Stahl, 2009; Fagiolini et al., 2012). This dual mechanism also confers sedative properties via antagonism at α1-adrenergic and H1-histaminergic receptors, supporting its use in patients with anxiety, sleep disturbances, or sexual disfunction (Fagiolini et al., 2012). Trazodone has shown efficacy comparable to TCAs and several second-generation antidepressants in treating symptoms of major depressive disorder (MDD) (Gartlehner et al., 2011; Cuomo et al., 2019). Its favorable safety profile and its great availability of formulations allow to personalize trazodone treatment according to patient profile characteristics (Cuomo et al., 2021).

Pharmacokinetically, trazodone is rapidly absorbed after oral administration, with immediate-release formulations achieving peak plasma levels within 0.5–2 h (DESYREL, 2024, OLEPTRO, 2024, Trittico, 2024). Extended-release formulations were developed to improve plasma concentration stability and minimize peak-related adverse effects (Karhu et al., 2011). Trazodone undergoes extensive hepatic metabolism, predominantly via cytochrome P450 (CYP) 3A4 (CYP3A4), which converts it into several metabolites, including m-chlorophenylpiperazine (mCPP)—its only known active metabolite (Rotzinger et al., 1998a). mCPP has high affinity for various serotonin receptors, including 5-HT2C, 5-HT3, 5-HT2A, 5-HT1B, and 5-HT1A (Caccia et al., 1981; Rotzinger et al., 1998a; Zalma et al., 2000; Kalgutkar et al., 2005). In humans, systemic levels of mCPP are typically below 10% of those of trazodone (Otani et al., 1997). Unlike trazodone, which primarily antagonizes 5-HT2A and 5-HT2C receptors, mCPP acts as an agonist, potentially modulating the net pharmacodynamic profile (Fiorella et al., 1995; Mihara et al., 2002). Trazodone’s metabolism involves not only CYP3A4 but also other hepatic enzymes such as CYP2D6, CYP2C19, and flavin-containing monooxygenases (FMOs), which contribute to secondary pathways like hydroxylation and N-oxidation (Rotzinger et al., 1998a). Despite its clinical relevance, detailed studies on the specific metabolic routes, enzyme contributions, and interspecies differences remain limited.

Research on central nervous system (CNS)-expressed CYP enzymes has underscored their relevance in modulating neurotransmitter dynamics, with significant implications for depressive disorders (Sheng et al., 2021). For instance, CYP2D6 in the brain is critical for the synthesis of serotonin from 5-methoxytryptamine and dopamine from tyramine, which influences serotonergic tone and dopaminergic signalling, both of which are fundamental to mood regulation and cognitive functions (Bromek et al., 2010; Bromek et al., 2011; Haduch et al., 2013; Haduch et al., 2015). Variability in CYP2D6 activity, whether due to genetic polymorphisms or drug-drug interactions, can influence serotonin and dopamine levels, potentially impacting antidepressant response and susceptibility to MDD (Ingelman-Sundberg et al., 2014; Penas-Lledo et al., 2015). In the substantia nigra, CYP2E1 participates in dopamine metabolism, and its role in producing reactive oxygen species (ROS) links it to neurodegenerative diseases like Parkinson’s, where oxidative stress exacerbates neuronal loss (Nissbrandt et al., 2001; Niazi Shahabi et al., 2003). CYP3A4, while predominantly hepatic, also plays a role in brain metabolism of psychotropic medications, affecting their local concentrations and possibly influencing both therapeutic and adverse effects (Ghosh et al., 2011; Sheng et al., 2021).

Despite its widespread use, current research on trazodone metabolic pathways, particularly CYP-mediated transformations, is limited and dated (Wen et al., 2008; Cuomo et al., 2019). While trazodone’s human metabolic pathways—principally CYP3A4-mediated N-dealkylation to mCPP and CYP2D6-mediated hydroxylation—are well characterized (Rotzinger et al., 1998a), early microsomal studies in rodents and rabbits demonstrate qualitatively different metabolic rates across species (Yamato et al., 1976). However, no peer-reviewed work has systematically quantified intrinsic clearance and mCPP formation in human versus rat hepatocytes. Given the significant role of CYP enzymes in both hepatic and CNS metabolism, filling this gap is essential to enhance the predictive power of preclinical *in vitro* models for human pharmacokinetics and drug–drug interaction risk (Sheng et al., 2021).

This study aims to further characterize the involvement of specific CYP isoforms in trazodone hepatic metabolism and to examine the inhibitory potential of trazodone and mCPP on major CYP enzymes. Comparative analyses across species and systems (microsomes, hepatocytes, and recombinant enzymes) were performed to clarify interspecies differences, with implications for translational pharmacokinetics and *in vitro*-to-*in vivo* extrapolation. 

## 2 Materials and methods

### 2.1 Hepatic metabolic stability

The metabolic stability of trazodone was assessed in cryopreserved hepatocytes from mouse, rat, and human sources to evaluate hepatic intrinsic clearance (CLint) and to characterize metabolic pathways. Trazodone hydrochloride was prepared at a final concentration of 10 μM, using William’s medium E supplemented with 8.9 mM glutamine and 89 mM Hepes as the incubation medium. Cryopreserved hepatocytes were pooled from mixed-gender human (H634/DCT, XenoTech/Bioreclamation IVT), pooled male mouse (CD-1, XenoTech), and pooled male Sprague-Dawley rat (XenoTech) sources, with a final concentration of approximately 0.5 million cells/mL. Incubations were conducted in duplicate at 37 °C for 180 min in a reaction volume of 200 μL. Standard deviations were not calculated due to small replicate size. To validate metabolic activity, testosterone (10 μM) and 7-hydroxycoumarin (5 μM) were used as phase I and phase II positive controls, respectively. An internal standard, trazodone D6 hydrochloride, was included at 500 ng/mL in the acetonitrile solution used for termination. Samples were collected at 12 designated time points (0, 5, 10, 15, 20, 30, 45, 60, 90, 120, 150, and 180 min) to capture detailed metabolic data, and reactions were terminated by adding acetonitrile at a 1:2 ratio to precipitate proteins. Following termination, samples were centrifuged. Supernatants were diluted with water to adjust the final organic solvent concentration (acetonitrile) to approximately 37%. Processed samples were analyzed using liquid chromatography-tandem mass spectrometry (LC-MS/MS) to monitor the disappearance of the parent compound over time (Bihan et al., 2015). Items were analyzed by LC-MS/MS on an Agilent HP1100 HPLC with a Synergi MAX RP (30 × 2 mm, 4 µm) column, using water + 0.1% formic acid (A) and acetonitrile + 0.1% formic acid (B) at 1.5 mL/min with a gradient from 5% B (0 min) to 95% B (1.5 min), held to 1.7 min, then returned to 5% B by 2.0 min, coupled to an AB Sciex API4000 mass spectrometer operating in positive MRM mode (372.0→176.1 for trazodone; 378.2→182.1 for D6; 289.2→109.0 for testosterone; 163.0→107.0 for 7-hydroxycoumarin).

CLint was calculated from the first-order elimination constant *k*, adjusted for the volume of incubation and hepatocyte count per reaction, and further normalized using a scaling factor for liver cells (expressed in million cells/g liver). Positive control reactions confirmed appropriate biotransformation activity in the hepatocytes, validating the experimental setup.

### 2.2 Metabolic profiling

The metabolic profile of trazodone was determined using *in vitro* incubations with rat and human liver microsomes, hepatocytes, and rat plasma samples. Trazodone was prepared at a final concentration of 8 μM. Microsomal incubations were conducted with pooled human and rat liver microsomes (Celsis IVT) using 0.6 mg/mL microsomal protein in 0.1 M phosphate buffer (pH 7.4). NADPH (1 mM) was added as a cofactor to initiate phase I metabolic reactions. Reactions were performed at 37 °C for 40 min with gentle shaking.

For hepatocyte incubations, pooled cryopreserved human hepatocytes (20-donor mix, Celsis) and male Sprague-Dawley rat hepatocytes (12-donor mix, Bioreclamation IVT) were used. Each incubation contained 1 million viable cells/mL in Celsis *InVitro* HI medium, at a pH of 7.4, and was conducted at 37 °C for 120 min under gentle agitation to facilitate metabolic activity.

In addition, rat plasma samples were collected at 0.25 and 6 h post-oral administration of trazodone at a dose of 30 mg/kg. Following incubation, all reactions were terminated by adding an equal volume of cold acetonitrile to precipitate proteins. Samples were then centrifuged at 13,000 x g for 10 min, and the supernatants were transferred to 96-well plates for analysis.

Processed supernatants (4 µL) were injected onto a Thermo U3000 UHPLC system coupled to a Thermo Q-Exactive Orbitrap MS equipped with a PDA detector. Separation was achieved on a Waters ACQUITY BEH C18 column (2.1 × 50 mm, 1.8 µm; 40 °C) at 0.50 mL min^-1^ using a gradient of 2 mM ammonium-formate (A) and acetonitrile (B): 95:5 (A:B) from 0 to 0.50 min, 50:50 at 3.00 min, 10:90 from 3.50 to 3.80 min, then back to 95:5 by 4.50 min. The Orbitrap operated in positive ESI mode (sheath/aux/sweep N_2_ 55/8/4 units; capillary 3.0 kV, 370 °C; auxiliary heater 550 °C) with full-scan m/z 100–1,000 at 35,000 FWHM (m/z 200) and DDI-MS/MS at 17,500 FWHM (7 Hz acquisition; IT 100 m; HCD 40 eV). External mass calibration and a 5 ppm extraction window were applied, and data were processed in Xcalibur 3.0.63/Compound Discoverer 1.0.

### 2.3 Identification of CYP isoforms using selective inhibitors

The role of specific CYP isoforms in trazodone metabolism was examined using selective inhibitors in human and rat liver microsomes. Trazodone (4 μM) was incubated with liver microsomal protein (0.4 mg/mL) in 0.1 M phosphate buffer (pH 7.4), with NADPH (1 mM) added to initiate phase I metabolic reactions. The incubation was conducted at 37 °C for 20 min with gentle shaking. Optimized concentrations of selective CYP inhibitors were introduced based on published studies (Turpeinen et al., 2005; Walsky et al., 2005; Turpeinen et al., 2006; Perloff et al., 2009). A summary of the CYP isoforms, corresponding inhibitors, and concentrations used is provided in Table 1. Reactions were terminated by adding an equal volume of acetonitrile to precipitate proteins, followed by centrifugation at 13,000 × g for 10 min.

**TABLE 1:** Selective cytochrome P450 (CYP) inhibitors used for reaction phenotyping in human and rat liver microsomes. Final concentrations were selected based on literature-reported specificity and efficacy.

CYP isoform	Selective inhibitor	Final concentration
CYP1A2	Fluvoxamine	1 μM
CYP2A6	Tranylcypromine	5 μM
CYP2B6	Ticlopidine	1 μM
CYP2C8	Montelukast	1 μM
CYP2C9	Sulfaphenazole	1 μM
CYP2C19	Fluconazole	10 μM
CYP2D6	Quinidine	1 μM
CYP2E1	Pyridine	60 μM
CYP3A4	Ketoconazole	1 μM

Metabolites were quantified under the same UHPLC-Orbitrap conditions described in § 2.2, ensuring consistent chromatography and MS performance across all inhibitor experiments. Metabolite levels in inhibitor-treated samples were compared to control samples (no inhibitor) to determine the extent of each CYP isoform involvement in metabolite production.

### 2.4 Identification of CYP isoforms using recombinant enzymes

To confirm the role of individual CYP isoforms in metabolizing trazodone, recombinant human and rat CYP enzymes were employed in separate incubations. Trazodone (4 μM) was incubated with each recombinant enzyme, including human CYP3A4, CYP3A5, CYP2D6, CYP2C19, CYP1A2, and FMO enzymes (FMO1 and FMO3), in a microsomal incubation buffer with NADPH as a cofactor at 37 °C for 40 min. Each reaction was terminated with acetonitrile, centrifuged, and the supernatant was analyzed by identical UHPLC-Orbitrap setup outlined in §2.2, allowing direct comparison of metabolite spectra across all *in-vitro* systems. The production of primary metabolites, such as M1, M2, M9, and M16, was monitored to determine which recombinant enzymes produced each metabolite. This procedure helped confirm the contributions of individual isoforms to specific biotransformation reactions.

### 2.5 Extrapolation to human using a predictive model

A predictive model was utilized to extrapolate the relative contributions of each CYP isoform in human liver to the overall metabolism of trazodone (Rostami-Hodjegan and Tucker, 2007). This model incorporated the known relative abundances of CYP isoforms in human liver tissue and was used to estimate the predominant pathways mediated by CYP3A4 and CYP3A5. The data from recombinant enzyme studies provided specific metabolite formation rates, which were adjusted for enzyme abundance to predict *in vivo* contributions. Additional extrapolation considered minor roles of CYP2C8, CYP2C19, CYP2D6, and FMO1/FMO3 based on enzyme activity data.

### 2.6 Inhibition of CYP enzymes by trazodone and mCPP

The last section of our study assessed the inhibitory effects of trazodone and its primary metabolite, mCPP, on the cytochrome P450 enzymes CYP1A2, CYP2A6, CYP2E1, and CYP3A4 in human and mouse hepatocytes. To evaluate the extent of enzyme inhibition, model probe substrates specific to each CYP enzyme were used: phenacetin for CYP1A2, coumarin for CYP2A6, chlorzoxazone for CYP2E1, and midazolam for CYP3A4. Known inhibitors served as positive controls, including furafylline for human CYP1A2, rutaecarpine for mouse CYP1A2, and tranylcypromine for CYP2A6, 4-methylpyrazole for CYP2E1, and ketoconazole for CYP3A4.

Cryopreserved human and mouse hepatocytes were obtained from Xenotech and Celsis, pooled from multiple male and female donors to increase experimental reliability. Prior to use, hepatocytes were stored in vapor-phase liquid nitrogen and then thawed, counted, and diluted in William’s Medium E, supplemented with glutamine and Hepes to reach a final concentration of approximately 0.5 million cells/mL.

Trazodone and mCPP were tested across an eight-point concentration-response curve, ranging from 100 μM to 0.1 μM, in triplicate. Positive controls were tested similarly but in singlicate. Due to the exploratory nature of these studies and absence of biological replicates, standard deviations were not calculated. Each test compound was dissolved in methanol (except 4-methylpyrazole, which was dissolved in water to prevent interference with CYP2E1 activity), and further serial dilutions were prepared in the same solvent. Methanol was used as a control at the highest solvent concentration.

Incubation plates were prepared by adding 5 μL of each test compound per well, followed by 1 μL of the respective probe substrate (200× stock solution). Substrates were prepared in acetonitrile (phenacetin and chlorzoxazone) or methanol (coumarin and midazolam) to achieve the final reaction concentrations. Finally, hepatocytes were added (195 μL per well), bringing the total incubation volume to 200 μL per well. Plates were maintained at 37 °C with shaking to support optimal enzyme activity.

After incubation, CYP enzyme activities were monitored by measuring the formation or disappearance of specific metabolites via high performance liquid chromatography-tandem mass spectrometry (HPLC-MS/MS). IC50 values, representing the concentration at which enzyme activity was inhibited by 50%, were calculated for each test compound. Samples (100 µL) were protein-precipitated with 200 µL acetonitrile containing internal standard, vortexed, centrifuged (20 min, 2 800 rpm), diluted 1 : 2 with 18% acetonitrile and 10–15 µL injected onto an Agilent HP1100 LC fitted with a Varian ODS-3 column (33 × 3 mm, 3 µm) and coupled to an AB Sciex API4000 triple-quadrupole (Turbo Ion Spray 650 °C; gas 45/45/25; MRM mode). Mobile phases were water +0.1% formic acid (A) and acetonitrile +0.1% formic acid (B) with 3 min gradients optimised for each probe substrate; analyte transitions were 152→110 (paracetamol), 161→133 (7-OH-coumarin), 184→120 (6-OH-chlorzoxazone) and 342→203 (1′-OH-midazolam).

## 3 Results

### 3.1 Hepatic metabolic stability

The CLint values revealed notable interspecies differences. In rat hepatocytes, trazodone exhibited a high clearance rate of 16.2 mL/min/g liver, indicating rapid metabolism. In mouse hepatocytes, the clearance rate was moderate at 4.14 mL/min/g liver, while in human hepatocytes, trazodone displayed a much slower clearance rate of 1.31 mL/min/g liver (Table 2).

**TABLE 2:** Metabolic stability data for trazodone in rat, mouse and human cryopreserved hepatocytes.

Species	M	R^2^	k (min^-1^)	CLint (µL/min/10^6^cells)	S (million cells/g liver)	CLint (mL/min/g liver)	Half life (min)	Test item % remaining last time point
Rat	0.5	0.971	0.075	150	108	16.2	9.22	0.521
Mouse	0.5	0.993	0.015	30.7	135	4.14	45.2	7.09
Human	0.5	0.974	0.006	11.2	118	1.31	124	35.8

### 3.2 Metabolic profiling

A comprehensive metabolic profiling study of trazodone identified 37 metabolites, labeled M1 through M37, across rat and human liver microsomes, hepatocytes, and rat plasma. Representative chromatograms are shown in Supplementary Figure S1, while the relative abundance (% of total LC-MS signal) for each metabolite across biological matrices is provided in Supplementary Table S1.

**FIGURE 1:**
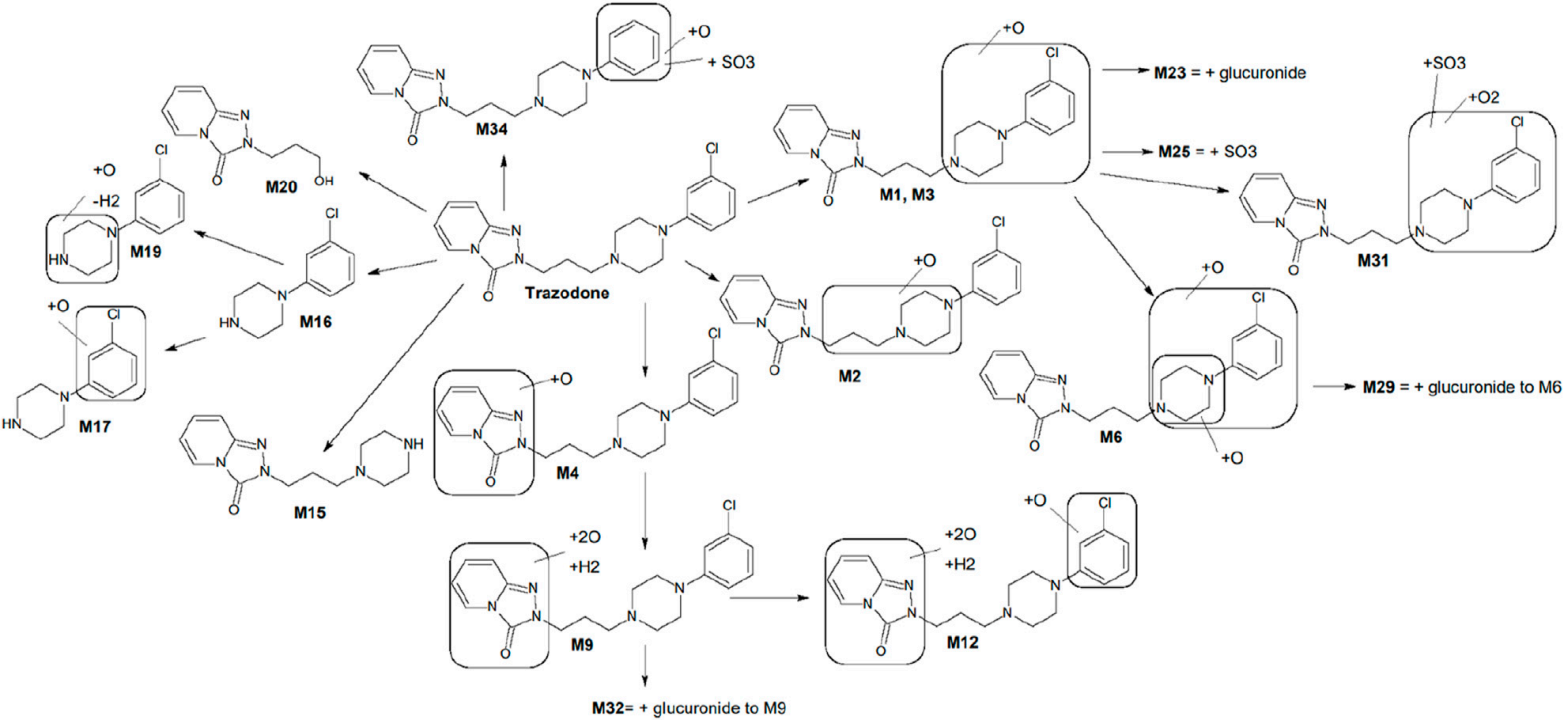
Proposed metabolic scheme of trazodone, constructed from *in vitro* incubations with human and rat liver microsomes, hepatocytes, and rat plasma. Key metabolites M1, M2, M9, M16, M17, and M20 were consistently observed in both human and rat systems. Metabolites such as M12, M23, and M25 showed markedly higher abundance in rat samples (particularly hepatocytes and plasma), whereas others displayed species-dependent variations in intensity. Full distribution and MS/MS characterization are detailed in Table 3 and Supplementary Figure S1.

Key metabolites common to both species included the oxidation product of chlorophenyl-propylpiperazine (M1), the oxidation product of propylpiperazine (M2), the dioxidation and hydrogenation derivative of triazole-pyridinone (M9), and m-chlorophenylpiperazine (mCPP, M16), which is formed via dealkylation by loss of propyltriazolopyridinone (C9H9N3O). Additionally, conjugated forms of M1, such as glucuronide (M23) and sulfo-conjugates (M25), were also identified (Figure 1). Among these, mCPP is the only metabolite known to retain pharmacological activity, particularly through its agonist action on serotonin receptors (Otani et al., 1997).

Incubations with liver microsomes and hepatocytes yielded a high number of metabolites across species, with distinct similarities noted in metabolite profiles between human and rat systems. Of the 37 total metabolites, 26 were observed in liver microsomes, 32 in hepatocytes, and 24 in rat plasma. UPLC-QE-Orbitrap-MS analysis revealed that M1 and M2 were the most prominent metabolites in both human and rat liver microsomes. M1 (chlorophenyl-propylpiperazine oxide), was consistently the dominant metabolite across species, followed closely by M2 (propylpiperazine oxide). In hepatocytes, additional conjugated metabolites such as M23 (glucuronide of M1) and M25 (sulfate of M1) were detected in rat samples, particularly in plasma.

The microsomal profiling showed that human and rat liver microsomes generated similar metabolite patterns, indicating a high degree of cross-species consistency. In human liver microsomes, M1 was the most abundant metabolite, followed by M2, M9 (triazole-pyridinone dioxidation and hydrogenation), and M16 (mCPP). Rat liver microsomes exhibited a comparable profile, with M1 again showing the highest abundance, along with significant levels of M2, M9, M16, M17 (chlorobenzene hydroxylation product), and M20 (alcohol derivative resulting from dealkylation at the opposite end of the molecule).

Further profiling in hepatocytes confirmed the presence of these metabolites with some variations in relative abundance. M12, another significant metabolite related to trazodone N-dealkylation, showed a higher presence in rat hepatocytes than in humans. In addition, conjugated metabolites such as M23 and M25 were observed at higher levels in rat hepatocytes and plasma, which aligns with the trend of increased conjugation activity in rat hepatocytes compared to humans. Metabolites in rat plasma matched qualitatively with the profiles obtained in microsomes and hepatocytes, particularly for M1, M2, M9, M16, M23, and M25. A summary of the key metabolites identified in rat and human systems, including retention time, molecular formula, and characteristic MS/MS fragment ions, is provided in Table 3.

**TABLE 3:** Summary of selected trazodone metabolites identified in rat and human liver microsomes and hepatocytes. For each metabolite, retention time (RT), molecular formula, observed and calculated m/z, and key MS/MS fragment ions are reported. These include both phase I metabolites (e.g., mono- and di-oxidation, dealkylation, dearylation) and representative bioactivation products.

Metabolite	Modification	RT (min)	Formula	Observed m/z	Calculated m/z	Representative Fragments (m/z)
M1	Oxidation in chlorophenyl-propylpiperazine	2.64	C19H22ClN5O2	3.881.531	3.881.535	253.1102, 225.0789, 176.0818
M2	Oxidation in propyl-piperazine	2.82	C19H22ClN5O2	388.153	3.881.535	370.1429, 356.1273, 278.1055
M6	Oxidation in chlorophenyl-piperazine + oxidation in propyl-piperazine	2.21	C19H22ClN5O3	4.041.485	4.041.484	372.1222, 294.1004, 225.0789
M9	2 x oxidation + hydrogenation in triazolopyridinone	2.89	C19H24ClN5O3	4.061.639	406.164	253.1295, 235.0997, 209.0840
M12	Hydroxylation in chlorobenzene + 2x oxidation + hydrogenation in triazolo-pyridinone	2.48	C19H24ClN5O4	422.159	422.159	390.1327, 253.1295, 210.0873
M15	Dearylation (loss of C6H3Cl)	1.42	C13H19N5O	2.621.661	2.621.662	176.0818, 148.0869, 133.0760
M16	Dealkylation (loss of C9H9N3O) = mCPP (meta-Chlorophenylpiperazine)	2.54	C10H13ClN2	1.970.841	197.084	180.0575, 166.0418, 154.0418
M17	Dealkylation (loss of C9H9N3O) + hydroxylation in chlorobenzene	1.15	C10H13ClN2O	213.079	2.130.789	211.0633, 196.0524, 178.1101
M19	Dealkylation (loss of C9H9N3O) + oxidation + dehydrogenation in piperazine	1.13	C10H11ClN2O	2.110.633	2.110.633	193.0527, 166.0418, 158.0838
M20	Dealkylation (loss of C10H11ClN2) + hydroxylation in propylchain	1.5	C9H11N3O2	1.940.925	1.940.924	176.0818, 148.0869, 133.0760

### 3.3 Identification of CYP isoforms using selective inhibitors

To identify specific CYP enzymes involved in the metabolism of trazodone, selective CYP inhibitors were applied in human and rat liver microsomal incubations. The inhibitors used included ketoconazole for CYP3A, fluconazole for CYP2C19, quinidine for CYP2D6, and fluvoxamine for CYP1A2, with each inhibitor employed at optimized concentrations to selectively inhibit its target enzyme (Turpeinen et al., 2005; Walsky et al., 2005; Turpeinen et al., 2006; Perloff et al., 2009).

In human liver microsomes (Table 4), the CYP3A inhibitor ketoconazole markedly reduced the formation of several key metabolites, notably M1 (chlorophenyl-propylpiperazine oxidation), M6 (mono-oxygenation product), M12 (N-oxidation product), M15 (hydroxylated derivative), M16 (mCPP), M17 (further chlorobenzene hydroxylation of mCPP), and M20 (alcohol product from dealkylation). Formation rates for these metabolites were reduced by 88%–100% compared to control samples without inhibitors, indicating that CYP3A4/5 plays a predominant role in their formation.

**TABLE 4:** The remaining percentage formation (as LC/MS peak areas) for the major trazodone metabolites in human liver microsomal incubations in the presence of CYP-selective inhibitors. ND = not detected.

Inhibitor / CYP	Conc. (µM)	Known IC50 (µM)^a^	M1	M2	M6	M9	M12	M15	M16	M17	M19	M20
Fluvoxamine / 1A2	1	0.1 – 0.2	100	117	126	96	97	139	117	87	ND	119
Tranylcypromine / 2A6	5	0.4 – 1.0	105	111	120	104	103	134	118	106	ND	122
Ticlopidine / 2B6	1	0.1 – 0.3	106	106	115	95	103	117	116	90	ND	118
Montelukast / 2C8	1	0.02 – 0.9^b^	95	100	88	108	80	83	98	117	ND	102
Sulphaphenazole / 2C9	1	0.2 – 0.4	106	107	101	109	147	107	108	103	ND	105
Fluconazole / 2C19	10	5.7 – 24	61	89	47	76	70	40	58	53	ND	50
Quinidine / 2D6	1	0.04 – 1.1	103	109	101	78	32	110	112	12	ND	100
Pyridine / 2E1	60	2.8 – 37	105	122	123	105	113	126	118	88	ND	121
Ketoconazole / 3A4	1	0.1 – 1.8^c^	12	70	4	61	0	5	5	5	ND	5

^a^ From Turpeinen et al. (2005) & references therein (Turpeinen et al., 2005).

^b^ From (Walsky et al., 2005; Perloff et al., 2009, Turpeinen et al., 2006; Walsky et al., 2005; Turpeinen et al., 2006; Perloff et al., 2009).

^c^ values for ketoconazole are highly dependent on the CYP3A4-substrate used.

The metabolite M9 (triazole-pyridinone dioxidation and hydrogenation) was less affected by ketoconazole but showed moderate inhibition by both fluconazole and quinidine, suggesting the involvement of multiple CYP enzymes in its formation, including CYP2C19, CYP2D6, and CYP3A4. This is similar to M12, which displayed a reduction down to 32% in formation with quinidine, indicating that CYP2D6 contributes alongside CYP3A4 to its production.

For M2 (propylpiperazine oxidation), ketoconazole produced a mild inhibition, reducing its formation to 70%, and other inhibitors had little to no effect, indicating that M2 formation may involve non-CYP pathways or other enzyme families, such as FMOs. This was consistent with the observed activity in recombinant FMO experiments (Table 5).

**TABLE 5:** Formation of trazodone major phase I metabolites catalyzed by recombinant human MAO, AOX and FMO enzymes (as LC/MS peak areas).

Enzyme	M1	M2	M6	M9	M12	M15	M16	M17	M19	M20
MAOA	12	129	0	0	0	0	28	0	0	0
MAOB	6	137	0	0	0	0	20	0	0	0
AOX	5	118	0	0	0	0	106	0	0	0
FMO1	59	7,599	0	0	0	0	6	0	0	0
FMO3	2	9,713	0	0	0	0	4	0	0	0

In rat liver microsomes (Table 6), the inhibition pattern was largely similar to that in humans, with ketoconazole reducing the formation of all major metabolites except M2, suggesting that CYP3A isoforms in rats play a similarly dominant role in metabolizing trazodone. However, unlike in human microsomes, quinidine had no significant effect on the formation of M9 and M12 in rats, which suggests that the rat CYP2D family may not contribute significantly to these pathways.

**TABLE 6:** The remaining percentage formation (as LC/MS peak areas) for the major trazodone metabolites in rat liver microsomal incubations in the presence of CYP-selective inhibitors.

Inhibitor / CYP	Conc. (µM)	Known IC50 (µM)^a^	M1	M2	M6	M9	M12	M15	M16	M17	M19	M20
Fluvoxamine / 1A2	1	0.1 – 0.2	85	118	99	70	72	90	126	65	104	133
Tranylcypromine / 2A6	5	0.4 – 1.0	92	120	102	98	101	96	130	99	101	140
Ticlopidine / 2B6	1	0.1 – 0.3	104	107	105	103	106	118	120	103	99	131
Montelukast / 2C8	1	0.02 – 0.9	97	106	93	116	106	102	100	98	85	96
Sulphaphenazole / 2C9	1	0.2 – 0.4	108	114	128	110	133	159	113	131	99	137
Fluconazole / 2C19	10	5.7 – 24	71	123	51	70	74	44	44	27	33	40
Quinidine / 2D6	1	0.04 – 1.1	113	110	117	98	93	147	115	101	108	121
Pyridine / 2E1	60	2.8 – 37	105	119	115	94	103	126	126	96	106	145
Ketoconazole / 3A4	1	0.1 – 1.8^b^	48	118	19	25	25	17	10	2	6	11

^a^ From Turpeinen et al. (2005) & references therein (Turpeinen et al., 2005).

^b^ values for ketoconazole are highly dependent on the CYP3A4-substrate used.

Fluvoxamine, a CYP1A2 inhibitor, had a mild inhibitory effect on M9, M12, and M18 in rat liver microsomes, reducing their formation to 65%–72% of control levels. This suggests a minor role for CYP1A2 in these pathways, specifically in rats. Notably, M2 formation in rat liver microsomes was unaffected by any of the CYP inhibitors tested, aligning with findings in human microsomes and further supporting the hypothesis that M2 may form via an alternative enzyme pathway, potentially involving FMOs or non-CYP mechanisms. M19, a minor downstream metabolite of mCPP, was undetectable in human liver microsomal incubations under all tested conditions (Table 4). In contrast, M19 was consistently observed in rat liver microsomes, and its formation was strongly inhibited by ketoconazole, suggesting a primary role for CYP3A enzymes in its biosynthesis in rodents. Fluconazole also moderately reduced M19 levels, indicating a possible secondary contribution from CYP2C19. Other CYP inhibitors had negligible effects (Table 6).

### 3.4 Identification of CYP isoforms using recombinant enzymes

To further elucidate the CYP isoforms involved in trazodone metabolism, recombinant CYP enzymes from both human and rat sources were employed, focusing on the production of major metabolites M1, M2, M9, and M16. Incubations with recombinant human CYP3A4 and CYP3A5 yielded the highest levels primarily of M1 and M16 and secondarily of M9 and M15, indicating a strong role for these isoforms in metabolizing trazodone. Among these, M1 formation was particularly prominent, following the order of CYP3A5 > CYP2D6 > CYP3A4 > CYP2C9 (Table 7).

**TABLE 7:** Formation of trazodone major phase I metabolites catalyzed by recombinant human CYP enzymes (as LC/MS peak areas).

CYP Isoform	M1	M2	M6	M9	M12	M15	M16	M17	M19	M20
CYP1A2	2998	223	0	2418	0	0	266	1	0	0
CYP2A6	74	138	0	8	0	0	8	0	0	0
CYP2B6	2213	148	0	988	0	0	112	0	10	0
CYP2C8	1689	167	0	286	0	26	327	0	0	0
CYP2C9	20190	151	0	3110	0	0	40	0	0	0
CYP2C19	6912	207	0	58433	0	180	189	0	0	0
CYP2D6	71022	157	0	48276	0	0	0	0	0	0
CYP2E1	119	144	0	49	0	0	16	12	0	0
CYP3A4	34798	3450	349	3949	66	6716	32991	581	0	236
CYP3A5	95557	317	36	5497	121	3343	16847	461	0	108

For M2, the primary enzyme contributing to its formation was CYP3A4, though both human FMO1 and FMO3 also facilitated M2 formation (Tables 5, 7), suggesting that its metabolic pathway may involve N-oxidation. This finding correlates with the low inhibition observed in microsomal studies when ketoconazole was used, indicating that CYP3A4 alone does not fully account for M2 formation. M16 (mCPP), resulting from N-dealkylation, was again most abundantly formed by CYP3A4, with secondary contribution from CYP3A5, in line with published evidence (Wen et al., 2008).

When recombinant rat CYP enzymes were used, M1 and M16 were again the most abundant metabolites (Table 8). CYP2C11, CYP2D4, and CYP3A9 produced M1, with CYP2C11 being the primary contributor, followed by CYP3A9 and CYP2D4. For M16, the highest formation was observed with CYP3A2 and CYP3A9, aligning with findings from human recombinant enzymes, further supporting the central role of CYP3A isoforms in N-dealkylation processes across species. These experiments confirmed the involvement of CYP3A4, CYP3A5, and CYP2D6 in the formation of major trazodone metabolites, with contributions from FMOs (Table 5).

**TABLE 8:** Formation of trazodone major phase I metabolites catalyzed by recombinant rat CYP enzymes (as LC/MS peak areas).

CYP Isoform	M1	M2	M6	M9	M12	M15	M16	M17	M19	M20
CYP1A2	4114	117	0	425	0	5	2151	113	3	232
CYP1B1	7638	84	0	19	0	22	724	0	0	94
CYP2C11	115280	161	0	1955	0	1210	6920	59	0	743
CYP2D4	36177	222	0	2827	0	184	3166	19	0	538
CYP2D18	27984	150	0	1543	0	134	1962	6	0	240
CYP3A2	191	1627	202	179	34	3368	45534	726	4	5761
CYP3A9	45963	1856	21	303	2	719	18577	76	0	1080

### 3.5 Extrapolation to human using a predictive model

A predictive model was used to extrapolate the contributions of each CYP isoform to trazodone metabolism in human liver, incorporating enzyme abundances based on established literature values for human liver tissue (Rostami-Hodjegan and Tucker, 2007). This model indicated that CYP3A4 and CYP3A5 would mediate the majority of trazodone metabolism, especially for the formation of M1 and M16 (mCPP). These findings are consistent with results obtained in rats, where ketoconazole markedly reduced the formation of M16 in liver microsomal incubations, confirming the predominant role of CYP3A isoforms in both species (Tables 4, 6).

The model further suggested that CYP2C8 might contribute to M1 formation as a minor pathway, while M9 was predicted to involve multiple CYP isoforms, including CYP2C9, CYP2C19, CYP2D6, and CYP3A, with CYP2C19 and CYP3A identified as the most relevant enzymes for this pathway. Additionally, M2 formation was projected to involve FMO1 and FMO3, consistent with the observed high abundance of M2 in recombinant FMO incubations (Table 5).

### 3.6 Inhibition of CYP enzymes by trazodone and mCPP

The inhibition potential of trazodone and mCPP on CYP1A2, CYP2A6, CYP2E1, and CYP3A4 was examined across a concentration-response curve in human and mouse hepatocytes.

In human hepatocytes, trazodone selectively inhibited CYP2E1 with an IC50 of 6.18 μM, demonstrating no inhibitory effects on CYP1A2, CYP2A6, or CYP3A4 up to the maximum concentration tested (100 μM). Furthermore, mCPP exhibited inhibitory activity in human hepatocytes, inhibiting both CYP2A6 and CYP2E1, with IC50 values of 29.8 μM and 6.46 μM, respectively. Notably, mCPP remained inactive against CYP1A2 and CYP3A4 in human hepatocytes, mirroring trazodone inactivity for these isoforms. These findings highlight a specific inhibitory action of trazodone and mCPP on CYP2E1, with mCPP also affecting CYP2A6 (Table 9), which do not directly impact *in vivo* activity, as the IC50 values measured *in vitro* are not reached by the unbound concentrations achieved in clinical practice (Fagiolini et al., 2023).

**TABLE 9:** Inhibition of cytochrome P450 enzymes by trazodone, mCPP and positive controls in human hepatocytes.

Compound ID	Isoform	IC50 value (µM)
Furafylline	CYP1A2	4.83
Trazodone	CYP1A2	>100
mCPP	CYP1A2	>100
Tranylcypromine	CYP2A6	1.72
Trazodone	CYP2A6	>100
mCPP	CYP2A6	29.8
4-Methylpyrazole	CYP2E1	0.83
Trazodone	CYP2E1	6.18
mCPP	CYP2E1	6.46
Ketoconazole	CYP3A4	0.05
Trazodone	CYP3A4	>100
mCPP	CYP3A4	>100

In mouse hepatocytes, trazodone inhibited CYP2E1 and CYP2A6, yielding IC50 values of 14.6 μM and 12.7 μM, respectively, indicating effective inhibition within this concentration range, while it resulted to be inactive up to 100 µM against CYP1A2 and CYP3A4. mCPP displayed significant potency against CYP2E1, with an IC50 of 1.88 μM, demonstrating a notably stronger inhibition than trazodone in this isoform (Table 10). In contrast, mCPP showed no activity against CYP1A2, CYP2A6, or CYP3A4 up to 100 μM in mouse hepatocytes. These results underscore a species-dependent variation in CYP inhibition profiles, with both trazodone and mCPP exhibiting stronger effects on CYP2E1 in mouse hepatocytes compared to human cells.

**TABLE 10:** Inhibition of cytochrome P450 enzymes by trazodone, mCPP and positive controls in CD1 mouse hepatocytes.

Compound ID	Isoform	IC50 value (µM)
Rutaecarpine	CYP1A2	11.8
Trazodone	CYP1A2	>100
mCPP	CYP1A2	>100
Tranylcypromine	CYP2A6	1.49
Trazodone	CYP2A6	12.7
mCPP	CYP2A6	>100
4-Methylpyrazole	CYP2E1	1.73
Trazodone	CYP2E1	14.6
mCPP	CYP2E1	1.88
Ketoconazole	CYP3A4	0.18
Trazodone	CYP3A4	>100
mCPP	CYP3A4	>100

Control experiments with known CYP inhibitors—furafylline, rutaecarpine, tranylcypromine, 4-methylpyrazole, and ketoconazole—yielded IC50 values consistent with literature and in-house data, affirming the assay validity. The observed inhibition of CYP2E1 by trazodone and mCPP in mouse hapatocytes suggests a potential involvement of this isoform in species-specific metabolic pathways.

## 4 Discussion

Despite trazodone’s widespread clinical use, detailed understanding of its biotransformation—particularly the generation and pharmacological relevance of mCPP—remains limited. This study provides a systematic characterization of hepatic metabolism across species, using hepatocytes, microsomes, and recombinant enzyme systems.

CLint data revealed notable interspecies differences, with rat hepatocytes showing over 12-fold higher clearance rates than human hepatocytes (Table 2). These findings are consistent with historical *in vivo* data and suggest a faster metabolic turnover in rodents, particularly for CYP3A-mediated pathways such as M1 and M16, as confirmed by inhibition data (Tables 7, 8). In contrast, clearance in human hepatocytes was significantly slower, supporting the potential for sustained plasma levels and highlighting the need for human-specific *in vitro* systems to predict pharmacokinetics (Jansson-Lofmark et al., 2020). For other metabolites formed via CYP2C19 and CYP2D6, the interspecies variation was less marked. The observed differences may help contextualize the broad range in trazodone plasma levels, ranging from 21.5 to over 2,200 ng/mL under standard dosing conditions (Ding et al., 2022).

The metabolic profiling of trazodone—performed through incubations with human and rat liver microsomes, hepatocytes, and rat plasma—provided a comprehensive view of its biotransformation pathways. A total of 37 distinct metabolites were detected, involving hydroxylation, N-dealkylation, and conjugation. This range of transformations supports a metabolic cascade where primary oxidative and dealkylation reactions generate intermediates subsequently subjected to phase II conjugation (Figure 1). The active metabolite mCPP was consistently identified in both human and rat systems. Other major metabolites, such as M1 and M9, were also present across species, indicating conserved oxidative pathways. However, differences in relative abundance—such as increased M12 levels in rat hepatocytes—highlighted species-specific trends for some metabolites. Thesedifferences highlight the importance of considering species-specific patways when interpreting preclinical pharmacokinetic data, as rodent models may exhibit faster metabolism or alternative pathways compared to humans. The proposed metabolic scheme integrates these findings, outlining sequential steps leading to mCPP and other key derivatives. Oxidative processes dominate the early stages, with both N-dealkylation and hydroxylation contributing to mCPP formation. Conjugated derivatives such as M23 and M25 were primarily observed in rat hepatocytes and plasma, reflecting enhanced phase II capacity in rodents. The concordance between rat plasma and *in vitro* systems supports the relevance of these models for characterizing metabolic pathways involved in trazodone disposition.

The use of selective CYP inhibitors in human and rat liver microsomes confirmed CYP3A4 as the primary enzyme responsible for converting trazodone to mCPP. Ketoconazole markedly reduced the formation of mCPP and related metabolites in both species, consistent with the established central role of CYP3A4 in trazodone metabolism (Caccia et al., 1981; Rotzinger et al., 1998a; Zalma et al., 2000; Kalgutkar et al., 2005). In human liver microsomes, partial inhibition of other metabolites by fluconazole and quinidine suggested secondary contributions from CYP2C19 and CYP2D6. This multi-enzyme involvement underscores the importance of pharmacogenetic variability: CYP2C19 and CYP2D6 polymorphisms may influence trazodone metabolic patterns, with potential implications for interindividual variability. Indeed, steady-state studies show that CYP2D6 poor metabolizers accumulate higher mCPP/trazodone ratios compared to extensive metabolizers (Mihara et al., 1997; Wen et al., 2008). A recent case report described hepatotoxicity in a patient with a CYP2D6 loss-of-function genotype, reinforcing the relevance of CYP2D6 in mediating mCPP exposure (Ferdinande et al., 2024). Moreover, a scoping review suggested that while CYP2D6, CYP3A4, and CYP1A2 variants have limited impact on trazodone pharmacokinetics, polymorphisms in the ABCB1 transporter may significantly affect drug exposure and clearance (Meulman et al., 2023). In rat liver microsomes, ketoconazole inhibition produced similar results, confirming that CYP3A isoforms also mediate trazodone metabolism in rodents. However, quinidine had negligible effects on mCPP formation in rats, likely reflecting species differences in CYP2D function or inhibitor sensitivity. Fluvoxamine slightly reduced the formation of M17 (a metabolite derived from mCPP), suggesting a minor role for CYP1A2 in this transformation. In contrast, M19 formation appeared unaffected. These species-dependent variations highlight the complexity of extrapolating preclinical metabolism data to humans, underling the importance of integrated approaches.

Recombinant enzyme studies confirmed that CYP3A4 and CYP3A5 exhibit the highest activity in forming mCPP. Alongside them, CYP2D6 and CYP2C19 also contributed to the formation of M9 and hydroxylated products. Consistent with prior literature, CYP2D6 was shown to mediate para-hydroxylation of mCPP to p-hydroxy-mCPP, a pathway inhibited by quinidine in microsomal assays (Rotzinger et al., 1998b). Notably, the M2 metabolite—only mildly affected by ketoconazole—was produced by both CYP3A4 and the flavin-containing monooxygenases FMO1 and FMO3, supporting an N-oxidation pathway. This dual enzyme involvement explains the weak effect of CYP inhibitors on M2 formation in microsomes. To further investigate species-specific contributions, Table 8 reports the formation of major phase I metabolites in rat recombinant CYP systems, expressed as LC/MS peak areas. Among the isoforms tested, CYP3A2 showed the most robust formation of M1, M4, and M16—supporting the key role of rat CYP3A orthologs in oxidative metabolism of trazodone. In contrast, CYP2D2 and CYP2C11 catalyzed minor metabolites such as M17 and M9 with lower efficiency, suggesting more selective or limited roles.

Finally, inhibition assays showed that trazodone and mCPP were found to inhibit CYP2E1 *in vitro*, with mCPP also showing inhibition of CYP2A6. However, the *in vitro* IC50 values were substantially higher than unbound plasma concentrations achieved clinically. Given trazodone’s established safety profile over decades of use,the observed *in vitro* inihibition occurred at concentrations unlikely to be reached *in vivo*, suggesting low clinical relevance. Nonetheless, this highlights the challenges associated with *in vitro*-to-*in vivo* extrapolation, particularly when estimating drug interaction potential based on IC50 values alone (Jansson-Lofmark et al., 2020). These findings suggest a low potential for CYP2E1-or CYP2A6 mediated interactions under therapeutic conditions. However, combined with the interspecies variability observed in mCPP clearance and formation, these findings further support the use of human-specific models in drug interaction risk assessment.

## 5 Conclusion

This study clarifies the complex biotransformation of trazodone, identifying CYP3A isoforms as the primary enzymes responsible for its metabolism, with auxiliary contributions from CYP2D6, CYP2C19, and FMOs. Interspecies differences were evident in overall metabolic clearance, as highlighted by markedly faster hepatic metabolism in rats compared to humans, and in the formation of CYP3A-derived metabolites such as mCPP. In contrast, the metabolic patterns associated with CYP2D6 and CYP2C19 were relatively consistent across species. While FMOs (FMO1 and FMO3) also contributed to specific metabolic steps, such as M2 formation, their activity was only assessed in recombinant human systems and thus cannot be compared across species. These findings indicate that cross-species extrapolation is most limited for CYP3A-mediated pathways and may affect the extrapolation of exposure estimates from preclinical models to humans.

Human-specific models and dosing treatment personalization remain open points for future research, considering administration in patients with multiple concurrent disorders and genetic CYP polymorphisms. Collectively, this study advances the understanding of trazodone metabolic pathways, and highlights the role of CYP-mediated metabolism in contributing to pharmacological profile of trazodone in the treatment of MDD.

## 6 Limitations

This study did not include a direct assessment of cytotoxicity or nonspecific enzyme inhibition in hepatocytes, microsomes, or recombinant systems, as such evaluations were beyond the study’s scope. Although the tested concentrations fall within commonly used *in vitro* ranges, the absence of dedicated viability assays or controls precludes a definitive assessment of potential off-target or cytotoxic effects, particularly at the upper end of the concentration range. These findings are derived exclusively from *in vitro* models and should be regarded as exploratory; these *in vitro* findings are exploratory and cannot be directly translated to clinical safety outcomes.

Additionally, replicate experiments were not performed at a scale sufficient to provide statistical error estimates for IC50 or CLint determinations. As a result, the reported values are presented without standard deviations and should be interpreted as qualitative or semi-quantitative indicators of metabolic activity and inhibition potential.

## Data Availability

The original contributions presented in the study are included in the article/Supplementary Material, further inquiries can be directed to the corresponding author.
