# Supplementary material for: Characterization of trazodone metabolic pathways and species-specific profiles
Source: Front Pharmacol. 2025 Sep 30;16:1636919. doi: 10.3389/fphar.2025.1636919 (PMC12518996; doi:10.3389/fphar.2025.1636919)
Supplement: Supplementary file 1 [file Supplementaryfile1.docx]

**Supplementary Material**

**
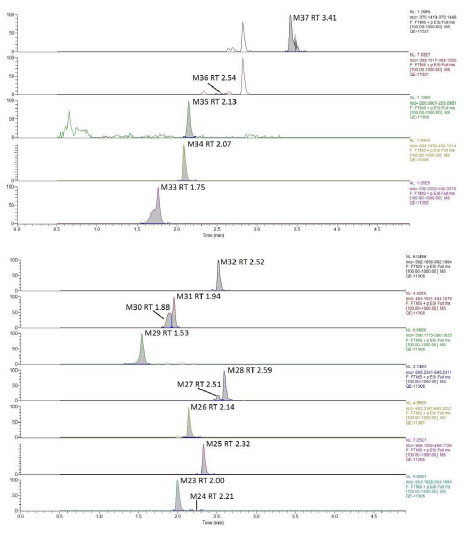
**

**
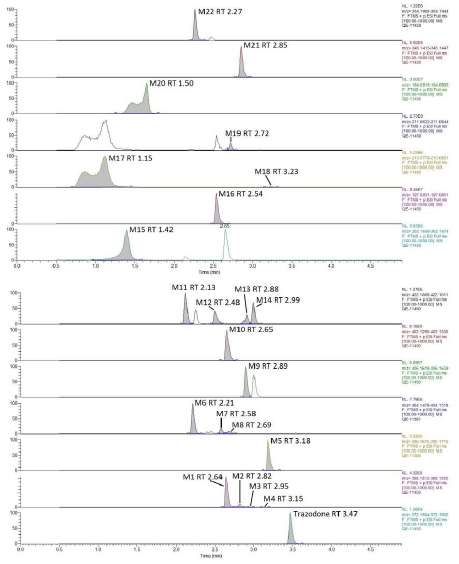
**

**Figure S1** - UPLC/QE-orbitrap-MS ion chromatograms for trazodone and its metabolites. All data is from human liver microsome incubation 40 min, except M5, M23-M25 is from human hepatocyte incubation 120 min, M29-M35 is from rat hepatocyte incubation 120 min, M6-M8 is from rat liver microsome incubation 40 min and M36 –M37 is from Rat number 8 plasma 0.25h sample.

|  | **Liver microsomes** | | **Hepatocytes** | | **Rat plasma** | |
| --- | --- | --- | --- | --- | --- | --- |
| **Metabolite** | **Human (%)** | **Rat (%)** | **Human (%)** | **Rat (%)** | **0.25h (%)** | **6h (%)** |
| **Trazodone** | 64.9 | 20.9 | 65.4 | 0.1 | 65.2 | 36.5 |
| **M1** | 20.7 | 41.7 | 5.3 | 0.2 | 1 | 1.3 |
| **M2** | 2 | 25.9 | 1.9 | 11.4 | 7.2 | 3.3 |
| **M3** | 0.3 | - | - | - | - | - |
| **M4** | 0.03 | 0.1 | 0.01 | 0.01 | 0.05 | 0.04 |
| **M5** | 0.01 | 0.01 | 0.2 |  |  |  |
| **M6** | 0.1 | 0.5 | 0.1 | 0.2 | 0.1 | 0.01 |
| **M7** | - | 0.1 | 0.01 | 0.4 | 0.1 | 0.02 |
| **M8** | - | 0.1 | - | - | - | - |
| **M9** | 2.4 | 0.7 | 8.7 | 1.7 | 7.9 | 8.2 |
| **M10** | 0.02 | - | 0.01 | - | 0.01 | - |
| **M11** | 0.1 | 0.04 | 0.1 | 0.01 | - | - |
| **M12** | 0.03 | 0.1 | 0.03 | 2.5 | 0.9 | 1.4 |
| **M13** | 0.02 | 0.01 | 0.05 | 0.1 | 0.2 | 0.3 |
| **M14** | 0.1 | 0.03 | 0.1 | 0.1 | 0.1 | 0.2 |
| **M15** | 0.3 | 0.8 | 0.2 | 0.7 | 0.1 | 0.1 |
| **M16** | 4.6 | 3.9 | 1.5 | 0.1 | 1 | 1.5 |
| **M17** | 1.1 | 1.5 | - | - | - | - |
| **M18** | 0.01 | 0.4 | 0.01 | 0.01 | 0.1 | 0.1 |
| **M19** | 0.01 | 0.7 | 0.01 | 0.6 | 0.1 | 0.1 |
| **M20** | 2.9 | 1.4 | 0.9 | 0.9 | - | - |
| **M21** | 0.01 | 0.01 | 0.01 | 0.05 | 0.2 | 0.1 |
| **M22** | 0.04 | 0.2 | 0.1 | 0.04 | 0.1 | 0.1 |
| **M23** | - | 0.01 | 9.7 | 70.7 | 3.1 | 33.3 |
| **M24** | - | - | 0.07 | - | - | - |
| **M25** | - | - | 5.5 | 6.2 | 12.1 | 12.7 |
| **M26** | 0.1 | 0.4 | - | 0.01 | - | - |
| **M27** | 0.1 | 0.2 | 0.01 | 0.05 | - | - |
| **M28** | 0.3 | 0.3 | 0.02 | 0.1 | - | - |
| **M29** | - | - | 0.01 | 1.1 | 0.05 | 0.02 |
| **M30** | - | - | - | 0.4 | - | - |
| **M31** | - | - | 0.04 | 0.8 | 0.04 | 0.04 |
| **M32** | - | - | - | 0.7 | - | - |
| **M33** | - | - | - | 0.2 | 0.04 | 0.2 |
| **M34** | - | - | 0.01 | 0.01 | - | - |
| **M35** | - | - | - | 0.01 | - | 0.1 |
| **M36** | - | - | - | - | 0.2 | - |
| **M37** | - | - | - | - | 0.1 | - |

**Table S1** - Metabolite profiles of trazodone from rat and human liver microsomes after 40 min incubation, hepatocytes after 120 min incubation, and rat plasma at 0.25 h and 6 h post-oral dosing.
